# Bis(η^5^-penta­methyl­cyclo­penta­dien­yl)cobalt(II)

**DOI:** 10.1107/S1600536809007971

**Published:** 2009-03-11

**Authors:** Meghan M. Clark, William W. Brennessel, Patrick L. Holland

**Affiliations:** a Department of Chemistry, University of Rochester, Rochester, NY 14627, USA

## Abstract

The crystal structure of the title compound, deca­methyl­cobaltocene, [Co(C_10_H_15_)_2_], has been determined. High-quality single crystals were grown from a cold saturated hexa­methyl­disiloxane solution. The structure is related to the manganese and iron analogs. The molecule has *D*_5*d*_ symmetry, with the Co atom in a crystallographic 2/*m* position. The cobalt–centroid(C_5_) distance is 1.71Å and the centroid(C_5_)–Co–centroid(C_5_) angle is 180°, by symmetry.

## Related literature

For the synthesis of the title compound and its electrochemical and magnetic properties, see: Robbins *et al.* (1982 ▶). For its formal potential and use as a reducing agent, see: Connelly & Geiger (1996 ▶). For the isotypic manganese and iron structures, see: Struchkov *et al.* (1978 ▶); Freyburg *et al.* (1979 ▶); Augart *et al.* (1991 ▶); Arrais *et al.* (2003 ▶).
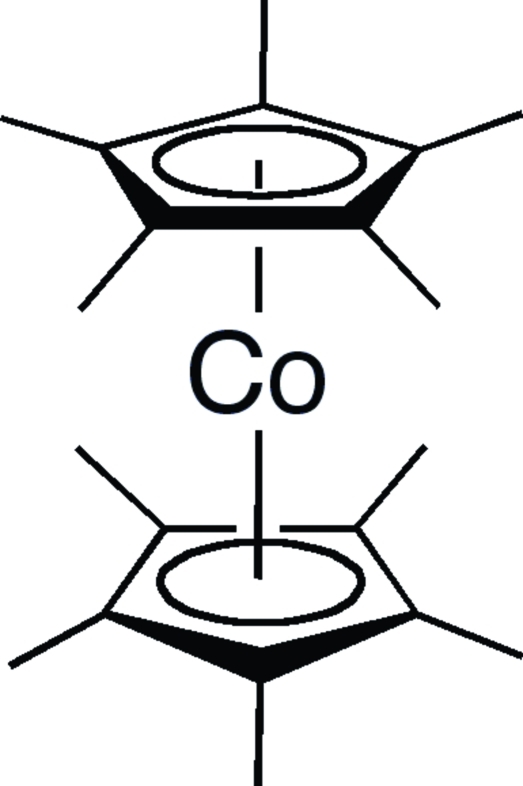


## Experimental

### 

#### Crystal data

[Co(C_10_H_15_)_2_]
*M* *_r_* = 329.37
Orthorhombic, 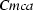

*a* = 15.0848 (16) Å
*b* = 11.5031 (12) Å
*c* = 10.0105 (10) Å
*V* = 1737.0 (3) Å^3^
*Z* = 4
Mo *K*α radiation
μ = 0.98 mm^−1^
*T* = 100 K
0.28 × 0.28 × 0.14 mm

#### Data collection

Bruker APEXII CCD diffractometer
Absorption correction: multi-scan (*SADABS*; Sheldrick, 2008*a* ▶) *T*_min_ = 0.771, *T*_max_ = 0.875
19672 measured reflections
2386 independent reflections
1903 reflections with *I* > 2σ(*I*)
*R*_int_ = 0.046

#### Refinement

*R*[*F* ^2^ > 2σ(*F* ^2^)] = 0.031
*wR*(*F* ^2^) = 0.089
*S* = 1.07
2386 reflections
84 parameters
All H-atom parameters refined
Δρ_max_ = 0.70 e Å^−3^
Δρ_min_ = −0.74 e Å^−3^

### 

Data collection: *APEX2* (Bruker, 2009 ▶); cell refinement: *SAINT* (Bruker, 2008 ▶); data reduction: *SAINT*; program(s) used to solve structure: *SHELXS97* (Sheldrick, 2008*b* ▶); program(s) used to refine structure: *SHELXL97* (Sheldrick, 2008*b* ▶); molecular graphics: *SHELXTL* (Sheldrick, 2008*b* ▶); software used to prepare material for publication: *SHELXTL*.

## Supplementary Material

Crystal structure: contains datablocks I, global. DOI: 10.1107/S1600536809007971/sj2590sup1.cif

Structure factors: contains datablocks I. DOI: 10.1107/S1600536809007971/sj2590Isup2.hkl

Additional supplementary materials:  crystallographic information; 3D view; checkCIF report

## Figures and Tables

**Table 1:** Selected bond lengths (Å)

Co1—C1	2.0914 (12)
Co1—C3	2.0956 (8)
Co1—C2	2.1113 (8)
C1—C2	1.4304 (12)
C1—C4	1.4961 (18)
C2—C3	1.4231 (12)
C2—C5	1.4935 (14)
C3—C6	1.4950 (13)

**Fig. 1.**
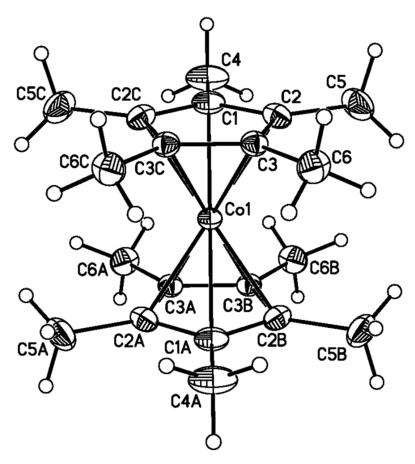
A displacement ellipsoid (50% probability) drawing of (I). The cobalt atom is in a crystallographic 2/m position.

## supplementary crystallographic information

### Comment

The structure of (I) has been conspicuously absent from the literature, despite its being a widely used reducing agent (Connelly & Geiger, 1996). Robbins and co-workers referred to a structural determination in 1982 (Robbins *et al.*, 1982), specifically its *D*_5_*_d_* symmetry and its similarity to the manganese analog. However, no structural data were presented. Attempts to grow single crystals from toluene and hexane, the latter from which Robbins reported having grown crystals, resulted in very poor quality specimens that were unsuitable for X-ray diffraction experiments. A cold (-38 ° C) saturated hexamethyldisiloxane solution of (I) afforded excellent crystals that resulted in a high quality structural determination.

The structure is isomorphous to that of decamethylferrocene (refcodes DMFERR, Struchkov *et al.*, 1978, DMFERR01, Freyburg *et al.*, 1979, DMFERR02, Arrais *et al.*, 2003) and the low temperature polymorph of decamethylmanganocene (refcodes DMCPMN01 and DMCPMN02, Augart *et al.*, 1991), for which the metal atoms are in crystallographic *2*/*m* positions.

### Experimental

All operations were performed under an inert atmosphere (dinitrogen). Hexamethyldisiloxane was stirred over CaH_2_ and vacuum transferred from sodium benzophenone ketyl. (I) was purchased from Sigma-Aldrich and used as is. Hexamethyldisiloxane (1 ml) was added to (I) (10 mg, 30 µmol), most of which dissolved over the course of a few hours at room temperature. After filtration through Celite, the filtrate was stored at -38 °C, resulting in dark yellow-brown crystals of (I) after a few hours.

### Refinement

Hydrogen atoms were found from the difference Fourier map and refined independently from their respective carbon atoms with individual isotropic displacement parameters.

### Crystal data

**Table:**

[Co(C_10_H_15_)_2_]	*F*(000) = 708
*M**_r_* = 329.37	*D*_x_ = 1.259 Mg m^−^^3^
Orthorhombic, *C**m**c**a*	Mo *K*α radiation, λ = 0.71073 Å
Hall symbol: -C 2bc 2	Cell parameters from 4000 reflections
*a* = 15.0848 (16) Å	θ = 3.0–37.5°
*b* = 11.5031 (12) Å	µ = 0.98 mm^−^^1^
*c* = 10.0105 (10) Å	*T* = 100 K
*V* = 1737.0 (3) Å^3^	Block, dark yellow-brown
*Z* = 4	0.28 × 0.28 × 0.14 mm

### Data collection

**Table:**

Bruker APEXII CCD diffractometer	2386 independent reflections
Radiation source: fine-focus sealed tube	1903 reflections with *I* > 2σ(*I*)
graphite	*R*_int_ = 0.046
φ and ω scans	θ_max_ = 38.0°, θ_min_ = 2.7°
Absorption correction: multi-scan (*SADABS*; Sheldrick, 2008a)	*h* = −25→25
*T*_min_ = 0.771, *T*_max_ = 0.875	*k* = −19→19
19672 measured reflections	*l* = −16→17

### Refinement

**Table:**

Refinement on *F*^2^	Primary atom site location: structure-invariant direct methods
Least-squares matrix: full	Secondary atom site location: difference Fourier map
*R*[*F*^2^ > 2σ(*F*^2^)] = 0.031	Hydrogen site location: inferred from neighbouring sites
*wR*(*F*^2^) = 0.089	All H-atom parameters refined
*S* = 1.07	*w* = 1/[σ^2^(*F*_o_^2^) + (0.0525*P*)^2^ + 0.2743*P*] where *P* = (*F*_o_^2^ + 2*F*_c_^2^)/3
2386 reflections	(Δ/σ)_max_ < 0.001
84 parameters	Δρ_max_ = 0.70 e Å^−^^3^
0 restraints	Δρ_min_ = −0.74 e Å^−^^3^

### Special details

**Table:**

Experimental. The crystal was examined under N_2_ and affixed to the end of a glass capillary with viscous oil, which protected the crystal during transfer to the cold stream.
Geometry. All e.s.d.'s (except the e.s.d. in the dihedral angle between two l.s. planes) are estimated using the full covariance matrix. The cell e.s.d.'s are taken into account individually in the estimation of e.s.d.'s in distances, angles and torsion angles; correlations between e.s.d.'s in cell parameters are only used when they are defined by crystal symmetry. An approximate (isotropic) treatment of cell e.s.d.'s is used for estimating e.s.d.'s involving l.s. planes.
Refinement. Refinement of *F*^2^ against ALL reflections. The weighted *R*-factor *wR* and goodness of fit *S* are based on *F*^2^, conventional *R*-factors *R* are based on *F*, with *F* set to zero for negative *F*^2^. The threshold expression of *F*^2^ > σ(*F*^2^) is used only for calculating *R*-factors(gt) *etc*. and is not relevant to the choice of reflections for refinement. *R*-factors based on *F*^2^ are statistically about twice as large as those based on *F*, and *R*- factors based on ALL data will be even larger.

### Fractional atomic coordinates and isotropic or equivalent isotropic displacement parameters (Å^2^)

**Table:**

	*x*	*y*	*z*	*U*_iso_*/*U*_eq_	
Co1	0.0000	0.0000	0.0000	0.01533 (7)	
C1	0.0000	−0.17732 (10)	0.04617 (12)	0.0228 (2)	
C2	−0.07677 (6)	−0.12416 (7)	0.10364 (8)	0.02149 (15)	
C3	−0.04749 (5)	−0.03551 (7)	0.19244 (8)	0.01843 (13)	
C4	0.0000	−0.27361 (12)	−0.05429 (14)	0.0348 (3)	
H4A	0.0000	−0.352 (3)	−0.013 (2)	0.045 (8)*	
H4B	−0.0486 (10)	−0.2711 (18)	−0.1133 (16)	0.063 (5)*	
C5	−0.17089 (8)	−0.15651 (11)	0.07606 (12)	0.0340 (2)	
H5A	−0.1798 (16)	−0.184 (2)	−0.0134 (18)	0.053 (6)*	
H5B	−0.2119 (12)	−0.0926 (16)	0.0778 (19)	0.059 (5)*	
H5C	−0.1896 (11)	−0.2189 (15)	0.1358 (16)	0.047 (4)*	
C6	−0.10544 (7)	0.04079 (10)	0.27607 (9)	0.02731 (18)	
H6A	−0.1134 (15)	0.0057 (13)	0.360 (3)	0.045 (6)*	
H6B	−0.1638 (11)	0.0562 (13)	0.2287 (15)	0.039 (4)*	
H6C	−0.0774 (11)	0.1158 (14)	0.2912 (14)	0.037 (4)*	

### Atomic displacement parameters (Å^2^)

**Table:**

	*U*^11^	*U*^22^	*U*^33^	*U*^12^	*U*^13^	*U*^23^
Co1	0.01811 (10)	0.01380 (9)	0.01409 (9)	0.000	0.000	−0.00032 (6)
C1	0.0353 (6)	0.0161 (4)	0.0170 (4)	0.000	0.000	−0.0004 (4)
C2	0.0245 (3)	0.0202 (3)	0.0198 (3)	−0.0054 (3)	−0.0013 (3)	0.0016 (3)
C3	0.0205 (3)	0.0180 (3)	0.0168 (3)	0.0002 (3)	0.0014 (2)	0.0006 (2)
C4	0.0653 (11)	0.0183 (5)	0.0206 (5)	0.000	0.000	−0.0029 (4)
C5	0.0290 (4)	0.0371 (5)	0.0359 (5)	−0.0147 (4)	−0.0069 (4)	0.0063 (4)
C6	0.0293 (4)	0.0292 (4)	0.0234 (4)	0.0056 (4)	0.0068 (3)	−0.0005 (3)

### Geometric parameters (Å, °)

**Table:**

Co1—C1^i^	2.0914 (12)	C2—C3	1.4231 (12)
Co1—C1	2.0914 (12)	C2—C5	1.4935 (14)
Co1—C3^ii^	2.0955 (8)	C3—C3^ii^	1.4328 (17)
Co1—C3	2.0956 (8)	C3—C6	1.4950 (13)
Co1—C3^i^	2.0956 (8)	C4—H4A	0.99 (3)
Co1—C3^iii^	2.0956 (8)	C4—H4B	0.942 (16)
Co1—C2^iii^	2.1113 (8)	C5—H5A	0.960 (18)
Co1—C2	2.1113 (8)	C5—H5B	0.962 (18)
Co1—C2^ii^	2.1113 (8)	C5—H5C	0.976 (17)
Co1—C2^i^	2.1113 (8)	C6—H6A	0.94 (2)
C1—C2	1.4304 (12)	C6—H6B	1.016 (16)
C1—C2^ii^	1.4304 (12)	C6—H6C	0.973 (16)
C1—C4	1.4961 (18)		
			
C1^i^—Co1—C1	180.0	C3—Co1—C2^i^	140.46 (3)
C1^i^—Co1—C3^ii^	113.16 (4)	C3^i^—Co1—C2^i^	39.54 (3)
C1—Co1—C3^ii^	66.84 (4)	C3^iii^—Co1—C2^i^	66.67 (3)
C1^i^—Co1—C3	113.16 (4)	C2^iii^—Co1—C2^i^	66.53 (5)
C1—Co1—C3	66.84 (4)	C2—Co1—C2^i^	180.0
C3^ii^—Co1—C3	39.98 (5)	C2^ii^—Co1—C2^i^	113.47 (5)
C1^i^—Co1—C3^i^	66.84 (4)	C2—C1—C2^ii^	108.12 (10)
C1—Co1—C3^i^	113.16 (4)	C2—C1—C4	125.94 (5)
C3^ii^—Co1—C3^i^	140.02 (5)	C2^ii^—C1—C4	125.93 (5)
C3—Co1—C3^i^	180.0	C2—C1—Co1	70.85 (6)
C1^i^—Co1—C3^iii^	66.84 (4)	C2^ii^—C1—Co1	70.85 (6)
C1—Co1—C3^iii^	113.16 (4)	C4—C1—Co1	124.99 (9)
C3^ii^—Co1—C3^iii^	180.0	C3—C2—C1	107.84 (8)
C3—Co1—C3^iii^	140.02 (5)	C3—C2—C5	126.09 (9)
C3^i^—Co1—C3^iii^	39.98 (5)	C1—C2—C5	126.07 (9)
C1^i^—Co1—C2^iii^	39.79 (3)	C3—C2—Co1	69.63 (5)
C1—Co1—C2^iii^	140.21 (3)	C1—C2—Co1	69.36 (6)
C3^ii^—Co1—C2^iii^	140.46 (3)	C5—C2—Co1	126.81 (7)
C3—Co1—C2^iii^	113.34 (3)	C2—C3—C3^ii^	108.08 (5)
C3^i^—Co1—C2^iii^	66.66 (3)	C2—C3—C6	126.09 (8)
C3^iii^—Co1—C2^iii^	39.54 (3)	C3^ii^—C3—C6	125.78 (5)
C1^i^—Co1—C2	140.21 (3)	C2—C3—Co1	70.82 (5)
C1—Co1—C2	39.79 (3)	C3^ii^—C3—Co1	70.01 (2)
C3^ii^—Co1—C2	66.67 (3)	C6—C3—Co1	126.88 (6)
C3—Co1—C2	39.54 (3)	C1—C4—H4A	112.8 (13)
C3^i^—Co1—C2	140.46 (3)	C1—C4—H4B	113.5 (11)
C3^iii^—Co1—C2	113.33 (3)	H4A—C4—H4B	107.0 (15)
C2^iii^—Co1—C2	113.47 (5)	C2—C5—H5A	112.8 (15)
C1^i^—Co1—C2^ii^	140.21 (3)	C2—C5—H5B	114.7 (11)
C1—Co1—C2^ii^	39.79 (3)	H5A—C5—H5B	100.5 (18)
C3^ii^—Co1—C2^ii^	39.54 (3)	C2—C5—H5C	110.1 (9)
C3—Co1—C2^ii^	66.67 (3)	H5A—C5—H5C	106.7 (16)
C3^i^—Co1—C2^ii^	113.33 (3)	H5B—C5—H5C	111.4 (14)
C3^iii^—Co1—C2^ii^	140.46 (3)	C3—C6—H6A	109.0 (11)
C2^iii^—Co1—C2^ii^	180.0	C3—C6—H6B	110.4 (9)
C2—Co1—C2^ii^	66.53 (5)	H6A—C6—H6B	112.4 (16)
C1^i^—Co1—C2^i^	39.79 (3)	C3—C6—H6C	110.7 (9)
C1—Co1—C2^i^	140.21 (3)	H6A—C6—H6C	107.2 (15)
C3^ii^—Co1—C2^i^	113.33 (3)	H6B—C6—H6C	107.2 (13)
			
C1^i^—Co1—C1—C2	22.38 (12)	C2^iii^—Co1—C2—C1	141.96 (6)
C3^ii^—Co1—C1—C2	80.81 (6)	C2^ii^—Co1—C2—C1	−38.04 (6)
C3—Co1—C1—C2	37.16 (5)	C2^i^—Co1—C2—C1	−59.99 (6)
C3^i^—Co1—C1—C2	−142.84 (5)	C1^i^—Co1—C2—C5	59.74 (11)
C3^iii^—Co1—C1—C2	−99.18 (6)	C1—Co1—C2—C5	−120.26 (11)
C2^iii^—Co1—C1—C2	−62.03 (10)	C3^ii^—Co1—C2—C5	158.44 (10)
C2^ii^—Co1—C1—C2	117.97 (10)	C3—Co1—C2—C5	120.46 (11)
C2^i^—Co1—C1—C2	180.0	C3^i^—Co1—C2—C5	−59.54 (11)
C1^i^—Co1—C1—C2^ii^	−95.59 (13)	C3^iii^—Co1—C2—C5	−21.56 (10)
C3^ii^—Co1—C1—C2^ii^	−37.16 (5)	C2^iii^—Co1—C2—C5	21.70 (8)
C3—Co1—C1—C2^ii^	−80.82 (6)	C2^ii^—Co1—C2—C5	−158.30 (8)
C3^i^—Co1—C1—C2^ii^	99.18 (6)	C2^i^—Co1—C2—C5	179.75 (9)
C3^iii^—Co1—C1—C2^ii^	142.84 (5)	C1—C2—C3—C3^ii^	−1.34 (8)
C2^iii^—Co1—C1—C2^ii^	180.0	C5—C2—C3—C3^ii^	178.27 (8)
C2—Co1—C1—C2^ii^	−117.97 (10)	Co1—C2—C3—C3^ii^	−60.38 (2)
C2^i^—Co1—C1—C2^ii^	62.03 (10)	C1—C2—C3—C6	−178.77 (9)
C1^i^—Co1—C1—C4	143.40 (12)	C5—C2—C3—C6	0.85 (14)
C3^ii^—Co1—C1—C4	−158.17 (3)	Co1—C2—C3—C6	122.20 (9)
C3—Co1—C1—C4	158.17 (3)	C1—C2—C3—Co1	59.04 (7)
C3^i^—Co1—C1—C4	−21.83 (3)	C5—C2—C3—Co1	−121.35 (9)
C3^iii^—Co1—C1—C4	21.83 (3)	C1^i^—Co1—C3—C2	142.61 (5)
C2^iii^—Co1—C1—C4	58.98 (5)	C1—Co1—C3—C2	−37.39 (5)
C2—Co1—C1—C4	121.01 (5)	C3^ii^—Co1—C3—C2	−118.44 (5)
C2^ii^—Co1—C1—C4	−121.01 (5)	C3^i^—Co1—C3—C2	−112.12 (6)
C2^i^—Co1—C1—C4	−58.99 (5)	C3^iii^—Co1—C3—C2	61.56 (5)
C2^ii^—C1—C2—C3	2.17 (13)	C2^iii^—Co1—C3—C2	99.14 (7)
C4—C1—C2—C3	−179.08 (11)	C2^ii^—Co1—C3—C2	−80.87 (7)
Co1—C1—C2—C3	−59.21 (6)	C2^i^—Co1—C3—C2	180.0
C2^ii^—C1—C2—C5	−177.44 (7)	C1^i^—Co1—C3—C3^ii^	−98.952 (19)
C4—C1—C2—C5	1.30 (17)	C1—Co1—C3—C3^ii^	81.049 (19)
Co1—C1—C2—C5	121.18 (9)	C3^i^—Co1—C3—C3^ii^	6.32 (2)
C2^ii^—C1—C2—Co1	61.38 (8)	C3^iii^—Co1—C3—C3^ii^	180.0
C4—C1—C2—Co1	−119.87 (12)	C2^iii^—Co1—C3—C3^ii^	−142.43 (3)
C1^i^—Co1—C2—C3	−60.72 (8)	C2—Co1—C3—C3^ii^	118.44 (5)
C1—Co1—C2—C3	119.28 (8)	C2^ii^—Co1—C3—C3^ii^	37.57 (3)
C3^ii^—Co1—C2—C3	37.98 (5)	C2^i^—Co1—C3—C3^ii^	−61.56 (5)
C3^i^—Co1—C2—C3	180.0	C1^i^—Co1—C3—C6	21.37 (9)
C3^iii^—Co1—C2—C3	−142.02 (5)	C1—Co1—C3—C6	−158.63 (9)
C2^iii^—Co1—C2—C3	−98.77 (5)	C3^ii^—Co1—C3—C6	120.32 (8)
C2^ii^—Co1—C2—C3	81.24 (5)	C3^i^—Co1—C3—C6	126.64 (8)
C2^i^—Co1—C2—C3	59.29 (5)	C3^iii^—Co1—C3—C6	−59.68 (8)
C1^i^—Co1—C2—C1	180.0	C2^iii^—Co1—C3—C6	−22.11 (9)
C3^ii^—Co1—C2—C1	−81.30 (6)	C2—Co1—C3—C6	−121.24 (10)
C3—Co1—C2—C1	−119.28 (8)	C2^ii^—Co1—C3—C6	157.89 (9)
C3^i^—Co1—C2—C1	60.72 (8)	C2^i^—Co1—C3—C6	58.76 (10)
C3^iii^—Co1—C2—C1	98.70 (6)		

Symmetry codes: (i) −*x*, −*y*, −*z*; (ii) −*x*, *y*, *z*; (iii) *x*, −*y*, −*z*.

## References

[bb1] Arrais, A., Diana, E., Gobetto, R., Milanesio, M., Viterbo, D. & Stanghellini, P. L. (2003). *Eur. J. Inorg. Chem.* pp. 1186–1192.

[bb2] Augart, N., Boese, R. & Schmid, G. (1991). *Z. Anorg. Allg. Chem.* **595**, 27–34.

[bb3] Bruker (2008). *SAINT* Bruker AXS Inc., Madison, Wisconsin, USA.

[bb4] Bruker (2009). *APEX2* Bruker AXS Inc., Madison, Wisconsin, USA.

[bb5] Connelly, N. G. & Geiger, W. E. (1996). *Chem. Rev.* **96**, 877–910. 10.1021/cr940053x 11848774

[bb6] Freyburg, D. P., Robbins, J. L., Raymond, K. N. & Smart, J. C. (1979). *J. Am. Chem. Soc.* **101**, 892–897.

[bb7] Robbins, J. L., Edelstein, N., Spencer, B. & Smart, J. C. (1982). *J. Am. Chem. Soc.* **104**, 1882–1893.

[bb8] Sheldrick, G. M. (2008*a*). *SADABS* University of Göttingen, Germany.

[bb9] Sheldrick, G. M. (2008*b*). *Acta Cryst.* A**64**, 112–122. 10.1107/S0108767307043930 18156677

[bb10] Struchkov, Yu. T., Andrianov, V. G., Sal’nikova, T. N., Lyatifov, I. R. & Materikova, R. B. (1978). *J. Organomet. Chem.* **145**, 213–223.

